# The efficacy and safety of sintilimab combined with chemotherapy as the first-line treatment for metastatic esophageal squamous cell carcinoma

**DOI:** 10.1097/MD.0000000000034794

**Published:** 2023-08-18

**Authors:** Zheng Zhao, Ming-Mei Yin, Wei-Feng Zhao, Chao-Jie Wang

**Affiliations:** a Department of Oncology, Henan Provincial People’s Hospital & People’s Hospital of Henan University, Zhengzhou, Henan, P.R. China.

**Keywords:** esophageal squamous cell carcinoma, immunotherapy, lactic dehydrogenase, nanoparticle albumin-bound paclitaxel, programmed death receptor 1, sintilimab

## Abstract

Immunotherapy is a new treatment option for patients with esophageal squamous cell carcinoma (ESCC). However, no study has investigated the efficacy and safety of sintilimab combined with nanoparticle albumin-bound paclitaxel (Nab-PTX) and platinum as first-line treatment for metastatic ESCC. In this retrospective study, eligible patients with metastatic ESCC were administered sintilimab plus Nab-PTX, cisplatin, or nedaplatin for up to 4 to 6 cycles. Subsequently, patients without progressive disease (PD) continued to receive sintilimab every 3 weeks as maintenance treatment until unacceptable toxicity, PD, withdrawal of consent, or for up to 2 years. The primary endpoint was the objective response rate (ORR) and the secondary endpoints were progression-free survival (PFS), overall survival (OS), disease control rate (DCR), and safety. A total of 22 patients diagnosed with metastatic ESCC were enrolled, 1 patient reached a complete response (CR), 15 patients achieved a partial response (PR), 4 patients had stable disease, and 2 had PD. The ORR was 72.7% (16/22) and the DCR was 90.9% (20/22). The time to response was 1.9 months (95% confidence interval [CI]:1.7–2.2 months). The median PFS was 8.9 months (95% CI, 7.1–10.7 months), and the median OS was 19.0 months. Exploratory biomarker analysis revealed that lactic dehydrogenase (LDH) was a potential marker for OS, and patients with high LDH levels had shorter mOS (13.0 months, 95% CI:7.5–18.5 months). Treatment-related adverse events (AEs) occurred in 21 patients (95.5%), most of which were grade 1 or 2. No treatment-related deaths occurred in this study. The results of this study suggested that sintilimab combined with Nab-PTX and platinum in patients with metastatic ESCC had a significantly high ORR and encouraging mPFS and mOS. LDH was a potential marker for OS, and the safety profile was manageable.

## 1. Introduction

Esophageal cancer (EC) is one of the most common malignancies worldwide, ranking seventh in terms of incidence (604,000 new cases) and sixth in terms of mortality (544,000 deaths) among all malignancies.^[1]^ Generally, EC can be classified into 2 main histological subtypes: esophageal squamous cell carcinoma (ESCC) and esophageal adenocarcinoma. Systemic chemotherapy is the first-line treatment. The 5-fluorouracil or paclitaxel combined with platinum is the standard first-line treatment for patients with metastatic or recurrent ESCC.^[2]^ The objective response rate (ORR) ranged from 37.2% to 58%, the median progression-free survival (PFS) and overall survival (OS) are approximately 4.8 to 7.9 months and 10.4 to 13.2 months, respectively.^[2–4]^ However, owing to the resistance and toxicity of chemotherapy, many patients are still not satisfied with the systemic treatment.

Platinum-based first-line chemotherapy provides limited survival benefits for patients with advanced ESCC. To achieve better efficacy, effective combination therapy with other therapies is urgently needed. More recently, immunotherapy, especially monoclonal antibodies against programmed death receptor 1 (PD-1) and its ligand (PD-L1), has provided new treatment options for patients with various tumors, including EC. When combined with immunotherapy, chemotherapy can increase the presentation of tumor antigens, promote the filtration of cytotoxic T lymphocytes, and improve the efficacy of immunotherapy.^[5]^ In the KEYNOTE-590 phase III study, pembrolizumab combined with cisplatin and fluorouracil as the first-line regimen for advanced or metastatic EC showed a significantly improved median OS and PFS.^[6]^

Sintilimab, a human IgG4 monoclonal antibody, was produced by Innovent Biologics and Eli Lilly. Sintilimab blocks the interaction of PD-1 with its ligands and helps recover the anti-tumor response of T-cells.^[7]^ Sintilimab showed antitumor effects in non-small cell lung cancer, hepatic cell cancer, classical Hodgkin lymphoma, etc.^[8–10]^ In a phase II study (ORIENT-2), 190 patients with advanced ESCC who failed to receive standard first-line chemotherapy were randomly assigned (1:1) to receive either chemotherapy (irinotecan or paclitaxel) or sintilimab. The median OS was 7.2 vs 6.2 months (*P* = .032; hazard ratio [HR] = 0.70; 95% confidence interval [CI], 0.50–0.97). Biomarker analysis showed that patients with high T-cell receptor clonality, low molecular tumor burden index, and neutrophil-to-lymphocyte ratio (NLR) < 3 at 6 weeks post-treatment showed the longest median OS.^[11]^ The ORIENT-15 is a multicenter, randomized phase 3 trial to evaluate the efficacy and safety of sintilimab versus placebo in combination with chemotherapy (cisplatin + paclitaxel or cisplatin + fluorouracil) as first-line treatment for unresectable ESCC. Sintilimab plus chemotherapy (S + C) versus chemotherapy (C) was superior for OS in all patients (median 16.7 vs 12.5 months, HR 0.63, 95% CI 0.51–0.78, *P* < .0001). PFS was superior with S + C vs C in all patients (median 7.2 vs 5.7 months, HR 0.56, 95% CI 0.46–0.68, *P <* .0001).^[12]^

Compared with paclitaxel, nanoparticle albumin-bound paclitaxel (Nab-PTX) may distribute tumors more efficiently and does not require dexamethasone pretreatment.^[13,14]^ But there is still no study to investigate sintilimab and Nab-PTX plus platin in the treatment of metastatic ESCC. In this retrospective study, we enrolled patients with metastatic ESCC who were treated with sintilimab and Nab-PTX plus cisplatin or nedaplatin. This study aimed to investigate the efficacy and toxicity of sintilimab combined with Nab-PTX and platinum as a first-line regimen in patients with metastatic ESCC.

## 2. Patients and methods

### 2.1. Patients and clinicopathological data

In this retrospective study, eligible patients had metastatic ESCC when diagnosed, and without previous systemic therapy. Patient data were retrospectively collected from October 2019 to September 2022. The main inclusion criteria were that the patients with histologically confirmed metastatic ESCC were male or female, aged from 18 to 75 years old, had at least 1 measurable lesion according to the Response Evaluation Criteria in Solid Tumors (RECIST; version 1.1), had an Eastern Cooperative Oncology Group (ECOG) performance status of <2, and adequate renal, hepatic, and hematologic function. The exclusion criteria included a history of active hepatitis B or C viral infection, autoimmune disease, ongoing systemic immunosuppressive therapy, concomitant secondary cancer, history of PD-1/PD-L1 therapy, or organ transplantation. Patients with a history of radiotherapy were excluded from the study. Written informed consent was obtained from all patients before entry into the study.

Data including sex, age, ECOG performance status, smoking status, complete blood count, tumor differentiation, PD-L1 expression, tumor location and lactate dehydrogenase (LDH) were collected from medical records and pathological reports. PD-L1 expression was assessed using PD-L1 IHC 22C3 pharmDx assay (Agilent Technologies, Santa Clara, CA, USA). Tumors positive for PD-L1 had a combined positive score (CPS) of ≥ 1. Peripheral blood test results, including lymphocytes, neutrophils, platelets, and LDH levels, were collected no more than 14 days before treatment. The variables of interest were the NLR, which was calculated by dividing the absolute number of neutrophils by the absolute number of lymphocytes, and the platelet-to-lymphocyte ratio (PLR), which was calculated by dividing the absolute number of platelets by the absolute number of lymphocytes.

The study was registered in the Chinese Clinical Trial Registry database (ChiCTR2100051909), and the protocol was reviewed and approved by the Chinese Ethics Committee of Registering Clinical Trials (ChiECRCT20210492). This study was conducted in accordance with the principles of the Declaration of Helsinki.

### 2.2. Treatment

The patients received sintilimab combined with Nab-PTX, cisplatin, or nedaplatin for up to 4 to 6 cycles (every 21 days). Each cycle included the intravenous administration of sintilimab (200 mg). Nab-PTX was intravenously infused for ≥ 30 minutes at 260 mg/m^2^ without antihistamine or corticosteroid premedication on day 1. Cisplatin or nadaplatin at a 60 to 75 mg/m^2^ dose on day 1 or divided into 3 days. After immunochemotherapy, patients without progressive disease (PD) continued to receive sintilimab every 3 weeks as maintenance therapy until unacceptable toxicity, PD, withdrawal of informed consent, or for up to 2 years.

### 2.3. Efficacy and toxicity

Baseline tumor sizes were collected less than 2 weeks before treatment. This study aimed to investigate the response rate and safety of sintilimab plus Nab-PTX and platinum therapy in patients with metastatic ESCC. The primary endpoint was ORR, which included complete response (CR) and partial response (PR). The secondary endpoints were PFS, OS, disease control rate (DCR), and safety. Assessment of tumor response was conducted using computed tomography every 1 or 2 cycles of treatment according to RECIST and every 2 months within the maintenance period until disease progression. Toxicities were graded according to the US Department of Health and Human Services Common Terminology Criteria for Adverse Events version 5.0.

### 2.4. Statistical analysis

Numeric data were presented as mean values and standard deviations (SDs). Categorical data were presented as frequency and percentage distributions. PFS was calculated from the beginning of the regimen until the day of disease progression, last follow-up day, or death. OS was calculated from the beginning of treatment until death from any cause or the last follow-up day. The Kaplan–Meier method was used to estimate PFS and OS, which were compared using the log-rank test. Cox regression models were used to analyze the relationship between the clinicopathological parameters and survival. The median and 95% CIs are also presented. Fisher exact test was used to assess the association of PD-L1, baseline NLR, and PLR with the ORR. All analyses were performed using SPSS software (version 22.0, Inc., Chicago, IL). *P* < .05 was considered statistically significant.

## 3. Results

### 3.1. Patient characteristics

Between October 2019 and July 2022, 26 patients were eligible for inclusion, and 2 patients declined to participate in this study. Two patients who underwent radiotherapy were excluded from the study. In total, 22 patients with metastatic ESCC were enrolled in this study. Baseline clinical and pathological characteristics of the patients are presented in Table 1. The median age of the patients was 63 years (range, 40–75). Six patients (27.3%) had an ECOG performance status score of 0, whereas 16 patients (72.7%) had an ECOG performance status score of 1. 40.9% (9/22) patients had a PD-L1 CPS of ≥ 10. The mean ± SD NLR and PLR were 2.91 ± 1.27, 155.74 ± 58.03, respectively. Using the mean value as the cutoff point, patients were divided into high or low NLR and PLR groups. In total, 54.5% (12/22), and 50% (11/22) of patients were divided into the high NLR and PLR groups, respectively.

**Table 1:** Baseline characteristics.

Baseline characteristics	N (%)
Age (yr)	
Mean	63
Range	40–75
Sex	
Male	17 (77.3)
Female	5 (22.7)
Smoking status	
Yes	11 (50)
No	11 (50)
ECOG PS	
0	6 (27.3)
1	16 (72.7)
Differentiation	
Well or moderately differentiated	11 (50)
Poorly differentiated	11 (50)
Location	
Upper	5 (22.7)
Middle	12 (54.5)
Lower	5 (22.7)
Platinum choice	
Cisplatin	9 (40.9)
Nedaplatin	13 (59.1)
PD-L1(CPS)	
<10	13 (59.1)
≥ 10	9 (40.9)
NLR	
<2.91	10 (45.5)
≥2.91	12 (54.5)
PLR	
<155.74	11 (50)
≥155.74	11 (50)
LDH (U/L)	
<250	13 (59.1)
≥250	4 (18.2)
Unknown	5 (22.7)

CPS = combined positive score, ECOG = eastern cooperative oncology group, LDH = lactic dehydrogenase, NLR = neutrophil to lymphocyte ratio, PD-L1, program death-ligand 1, PLR = platelet to lymphocyte ratio, PS = performance status.

### 3.2. Efficacy and survival

The data cutoff date was September 1^st^, 2022; 5 patients died of cancer, and the other 17 patients were still alive. The median treatment duration was 7.9 months (range 3.5–26 moths). The median number of treatment cycles of sintilimab were 11.3 (range, 5–33.8), and 7 patients remained on sintilimab treatment. A total of 15 patients stopped sintilimab treatment. Disease progression is the most common reason for treatment discontinuation (13/15), and 2 patients were of adverse events (AEs) (2/15) (one cause of immunotherapy related pneumonitis, and 1 cause of tracheoesophageal fistula). The median treatment cycles of chemotherapy were 5.1 (range, 3–6).

**Figure 1.**
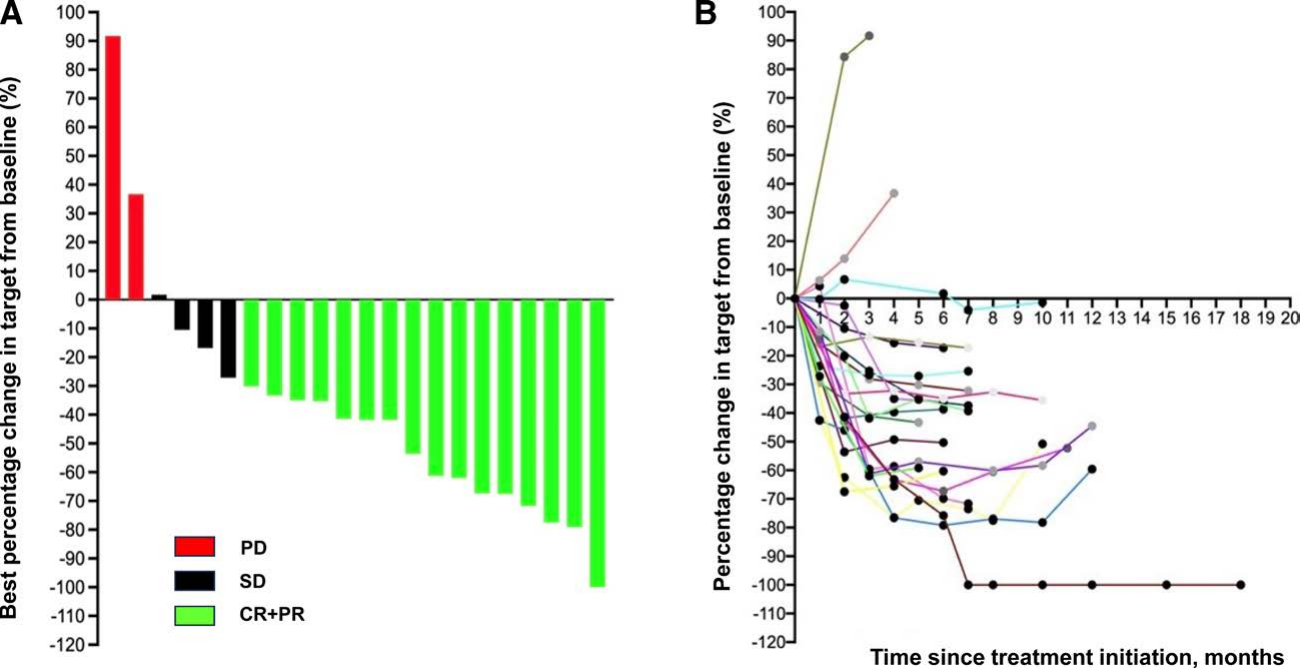
Response to sintilimab combined with chemotherapy as the first-line regimen for patients with metastatic ESCC. (A) Best change of target lesions compared with baseline. Each bar represents an individual patient. (B) Longitudinal change of target lesions compared with baseline. ESCC = esophageal squamous cell carcinoma.

Nineteen patients had a decrease in the sum of the target lesions, whereas 3 patients experienced an increase in the number of target lesions, as shown in Figure 1. According to the RECIST 1.1 guidelines, 1 patient achieved CR, 15 patients achieved PR, 4 patients achieved SD, and 2 patients had PD. The ORR was 72.7% (16/22) and the DCR was 90.9% (20/22) (Table 2). The time to respond was 1.9 months (95% CI:1.7–2.2 months).

**Table 2:** Activity of sintilimab combined with chemotherapy as the first-line regimen for metastatic ESCC patients.

Efficacy variable	Number of patients (%)
Best overall response	
Complete response	1 (4.5)
Partial response	15 (68.2)
Stable disease	4 (18.2)
Progressive disease	2 (9.1)
Objective response rate	16 (72.7)
Disease control rate	20 (90.9)

ESCC = esophageal squamous cell carcinoma.

**Figure 2.**
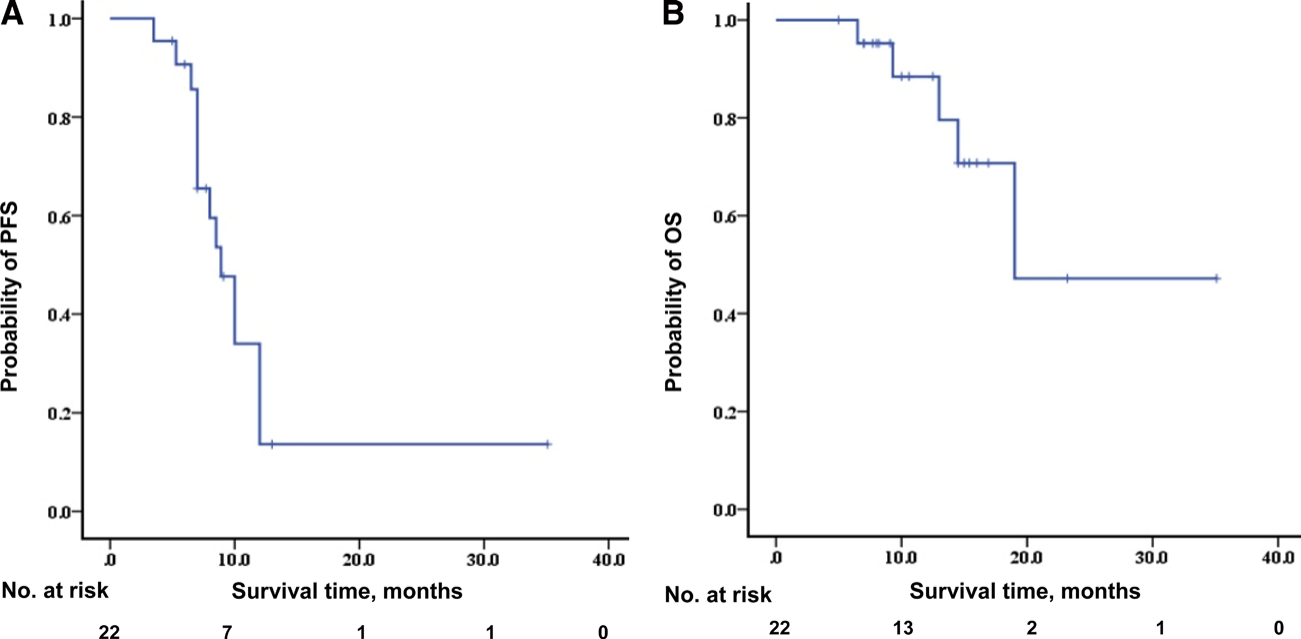
Kaplan–Meier curve for progress free survival (PFS) (A) and overall survival (OS) (B). The median PFS was 8.9 mo, median OS was 19.0 mo.

The median follow-up period was 11.6 months (ranges:5–35.1). The median PFS was 8.9 months (95% CI, 7.1–10.7 month), and 1 patient has 35.1 months PFS. The median OS was 19.0 months (Fig. 2).

### 3.3. Toxicity

All the 22 patients were included in the safety analysis (Table 3). Treatment-related AEs occurred in 21 patients (95.5%), most of which were grade 1 or 2. Grade 3 or 4 AEs occurred in 10 patients (45.5%), and there were no treatment-related deaths in this study. The most common AEs were hematological toxicity, including leukopenia (86.4%), neutropenia (72.7%), and thrombocytopenia (50%). Nausea and vomiting occurred in 63.6% of the patients. The most common grade 3 or 4 AEs were leukopenia (18.2%), neutropenia (13.6%), and thrombocytopenia (13.6%), respectively. These AEs were mainly related to chemotherapy. Immune-related AEs included pruritus (22.7%), hypothyroidism (9.1%), and pneumonitis (4.5%). One patient discontinued sintilimab treatment because of pneumonitis. No treatment-related deaths occurred during the study.

**Table 3:** Drug-related adverse events.

Adverse events	Grade 1 or 2 n (%)	Grade 3 or 4 n (%)
Leukopenia	15 (68.2)	4 (18.2)
Neutropenia	13 (59.1)	3 (13.6)
Nausea/vomiting	12 (54.5)	2 (9.1)
Thrombocytopenia	8 (36.4)	3 (13.6)
Peripheral neuropathy	6 (27.3)	0
Anemia	5 (22.7)	0
Pruritus	5 (22.7)	1 (4.5)
Fatigue	5 (22.7)	0
Constipation	4 (18.2)	0
Diarrhea	3 (13.6)	0
Pyrexia	3 (13.6)	0
Elevated transaminase	3 (13.6)	0
Hypothyroidism	2 (9.1)	0
Rash	2 (9.1)	0
Increased bilirubin	1 (4.5)	0
Increased creatinine	1 (4.5)	0
Pneumonitis	1 (4.5)	0

### 3.4. Biomarker analysis

**Figure 3.**
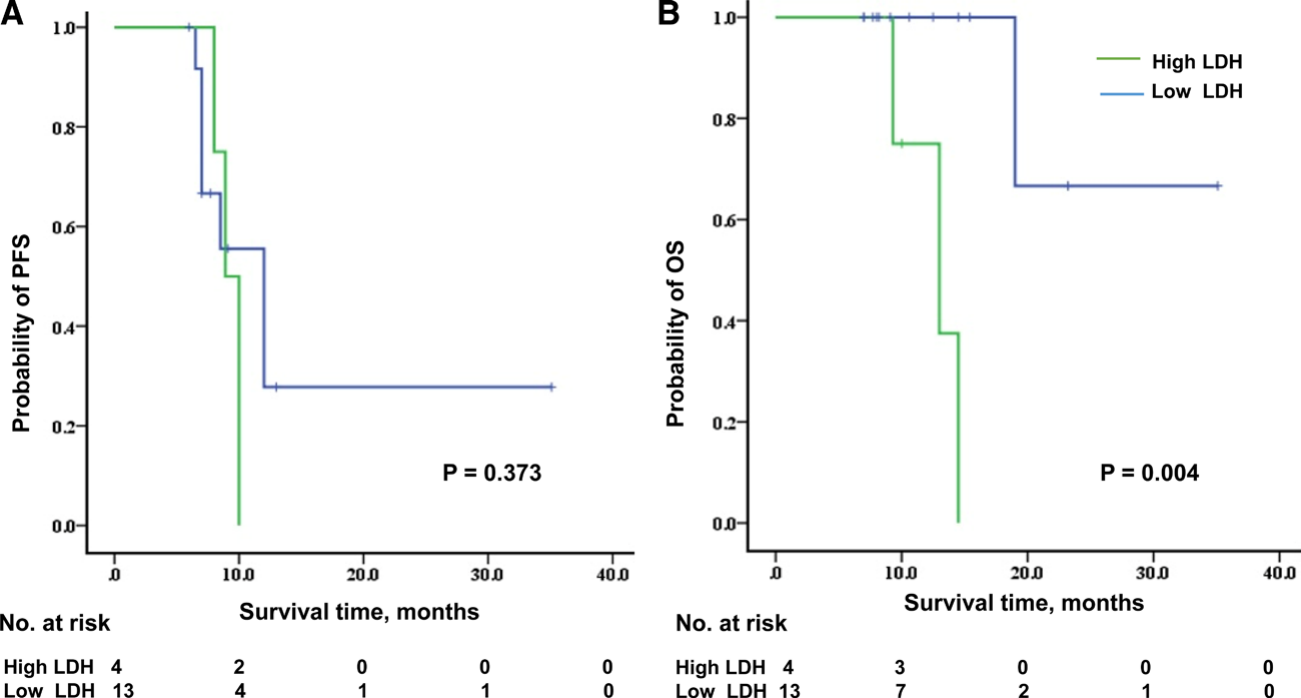
The relationship of lactic dehydrogenase (LDH) and patient survival. (A) The median progress free survival (mPFS) was not significantly different between patients with high and low LDH levels (8.9 vs 12.0 mo, *P* = .373). (B) High LDH levels were related to worse patient overall survival (OS). The mOS for patients with high LDH was 13 months (95%CI: 7.5–18.5 mo), but the mOS for patients with low LDH was not reached (*P* = .004). CI = confidence interval.

We investigated predictive biomarkers for patient survival, including age, gender, smoking status, ECOG performance status, PD-L1, LDH, NLR, and PLR. Univariate Cox regression analysis revealed that high LDH levels were associated with poor OS. The mOS was 19.0 months for all patients. The mOS for patients with high LDH was 13 months (95%CI: 7.5–18.5 months), but the mOS for patients with low LDH was not reached (*P* = .004) (Fig. 3). The mPFS was not significantly different between patients with high and low LDH levels (8.9 vs 12.0 months, *P* = .373). LDH was not an independent prognostic biomarker in multivariable Cox regression analysis. Other markers, including age, gender, smoking status, ECOG performance status, PD-L1, NLR, and PLR, were not associated with patient survival (data not shown). Tissue samples from 40.9% (9/22) had a PD-L1 CPS ≥ 10 and 59.1% (13/22) had a PD-L1 CPS < 10. The ORR was 88.9% (8/9) in PD-L1 CPS ≥ 10 and 61.5% (8/13) in PD-L1 CPS < 10. Although the ORR was higher in the PD-L1 CPS ≥ 10 group than in the PD-L1 CPS < 10 groups, the difference was not significant (*P* = .18). The ORR was 66.7% (8/12) in patients with a high NLR and 80% (8/10) in those with a low NLR (*P* = .42). The ORR was 63.6% (7/11) in the high PLR group and 81.8% (9/11) in the low PLR group (*P* = .32). No significant difference in clinical response rate was found between patients with high and low PD-L1 expression, NLR, or PLR.

## 4. Discussion

In this retrospective study, we investigated the efficacy and safety of sintilimab plus Nab-PTX and platinum as a first-line regimen in 22 patients with metastatic ESCC. The results showed that the ORR and DCR were 72.7% (16/22) and 90.9% (20/22), the median PFS was 8.9 months, and the median OS was 19.0 months. No new immunotherapy-related AE or treatment-related deaths occurred. To the best of our knowledge, this is the first study to report a combination of sintilimab with Nab-PTX and platinum as first-line treatment for patients with unresectable ESCC.

Immunotherapy targeting PD-1/PD-L1 has made breakthroughs in the recent years. In our study, sintilimab plus Nab-PTX and platinum could increase the ORR (72.7%), DCR (90.9%), and median PFS (8.9 months) compared with previous chemotherapy. This finding is consistent with the results of other studies. As a neoadjuvant regimen, sintilimab combined with cisplatin and Nab-PTX in patients with locally advanced ESCC had an ORR of 67% (20/30) and DCR of 97% (29/30).^[15]^ As we were preparing our manuscript, the results of the ORIENT-15 have been published. Sintilimab with chemotherapy (cisplatin plus paclitaxel or cisplatin plus 5-fluorouracil) showed better mOS and mPFS than placebo and chemotherapy in all patients (16.7 vs 12.5 months, HR:0.63, 95% CI: 0.51–0.78, *P* < .001) and (7.2 vs 5.7 months, HR:0.56, 95% CI:0.46–0.68, *P* < .001). The ORR was significantly higher in the sintilimab group than in the chemotherapy group (66% vs 45%, *P* < .001).^[12]^ Our study and other published data showed that anti-PD-1 immunotherapy combined with cytotoxic drugs had encouraging anti-tumor activity as the first-line treatment for patients with metastatic ESCC.

Nab-PTX is a novel solvent-free drug that uses albumin to deliver paclitaxel. A previous study showed that Nab-PTX could reach tumors more efficiently with enhanced accumulation than solvent-based paclitaxel.^[14]^ Nab-PTX in combination with cisplatin as a first-line regimen for ESCC resulted in an ORR of 50.0% to 60.6%, DCR of 81% to 87.9%, median PFS of 6.1 to 6.2 months and median OS of 12.5 to 15.5 months.^[16,17]^ In our study, sintilimab plus Nab-PTX and platinum had a higher ORR (72.7%), longer mPFS (8.9 months), mOS (19.0 months) than previous data. Accumulating evidence has shown that the combination of Nab-PTX and PD-1 inhibitor in ESCC exerts an obvious synergistic effect with lower toxicity.^[18,19]^ However, the underlying mechanism remains unknown, possibly because Nab-PTX can improve the efficacy of PD-1 inhibitors by regulating various immune functions.^[20,21]^ More recently, it was found that the synergistic mechanism may lie in depleting the Treg ratio to activate an immune response.^[22]^ Furthermore, the use of Nab-PTX plus PD-1 inhibitors has several unique benefits. For instance, the infusion conditions required for Nab-PTX or PD-1 inhibitors are simple and convenient, with no premedication requirement, decreased risk of hypersensitivity, and are important considerations for patient satisfaction.

From a metabolic perspective, one of the features of malignant cells is the production of a large amount of lactic acid, LDH is a key enzyme that is required for the conversion of pyruvate to lactic acid. A large amount of lactic acid accumulation in the tumor microenvironment can lead to the differentiation of Treg cells, resulting in a suppressed microenvironment.^[23,24]^ A previous retrospective analysis of 906 patients demonstrated that a high LDH level was associated with shorter survival, more advanced stage, and more distant metastasis in the era of chemotherapy.^[25]^ More recently, in another single-center retrospective study of 614 patients with ESCC who received chemoradiotherapy, patients in the high LDH group had significantly shorter PFS and worse OS than those in the low LDH group.^[26]^ Even in the era of immunotherapy, increasing evidence has shown that LDH levels are associated with patient survival.^[27,28]^ In this study, the mOS for patients with high LDH was 13 months (95%CI:7.5–18.5 months), but the mOS for patients with low LDH was not reached (*P =* .004), which suggested that the LDH could be a potential prognostic biomarker. This finding is in line with those of previous ESCC studies.^[29]^ In clinical practice, a serum LDH test would be helpful in selecting patients before initiating immunotherapy, and has the advantages of lower expense and rapid results.

PD-L1 expression in tumors and/or tumor-associated immune cells is a useful predictive biomarker, but the results remain controversial. In advanced ESCC, PD-L1 is a predictive biomarker for pembrolizumab,^[6]^ but the effectiveness of camrelizumab or tislelizumab was not associated with PD-L1 expression.^[30,31]^ In the present study, the ORR was 88.9% (8/9) in PD-L1 CPS ≥ 10 patients, and 61.5% (8/13) in PD-L1 CPS < 10 patients. Although the ORR was higher in the PD-L1 CPS ≥ 10 group than in the PD-L1 CPS < 10 group, the difference was not significant (*P* = .18). Biomarkers related to sintilimab efficacy require further study, and dynamic monitoring may be important.

Our study had some limitations. First, this was a retrospective study conducted at a single hospital, which may have caused a bias. Second, the study used a single arm, and the sample size was small, which may have limited the statistical power. Third, LDH as a predictor of OS was only significant through univariant Cox regression analysis; one of the possible reasons was the small sample size. Finally, the potential synergistic mechanisms of these 2 drugs should be elucidated. Although patients treated with sintilimab combined with Nab-PTX and platinum had a higher ORR and better median PFS than those in previous studies, the efficacy and safety need to be further elucidated in a larger phase III study.

In conclusion, the results of this study suggest that sintilimab combined with Nab-PTX and platinum for patients with metastatic ESCC had a significantly high ORR and an encouraging median PFS and OS. LDH was a potential marker of patient survival. This regimen warrants further investigation for treatment of metastatic ESCC.

## Acknowledgments

The authors wish to thank all the study participants and their families.

## Author contributions

**Conceptualization:** Wei-Feng Zhao, Chao-Jie Wang.

**Data curation:** Zheng Zhao, Ming-Mei Yin, Chao-Jie Wang.

**Formal analysis:** Zheng Zhao, Ming-Mei Yin, Wei-Feng Zhao.

**Investigation:** Zheng Zhao, Ming-Mei Yin, Wei-Feng Zhao, Chao-Jie Wang.

**Writing – original draft:** Zheng Zhao.

**Writing – review & editing:** Chao-Jie Wang.
